# Comparative Transcriptomic Analysis Reveals Candidate Genes and Pathways Involved in Larval Settlement of the Barnacle *Megabalanus volcano*

**DOI:** 10.3390/ijms18112253

**Published:** 2017-10-27

**Authors:** Guoyong Yan, Gen Zhang, Jiaomei Huang, Yi Lan, Jin Sun, Cong Zeng, Yong Wang, Pei-Yuan Qian, Lisheng He

**Affiliations:** 1 Department of Life Sciences, Institute of Deep-sea Science and Engineering, Chinese Academy of Sciences, Sanya 572000, China; yanguoyong@idsse.ac.cn (G.Y.); huangjiaomei@idsse.ac.cn (J.H.); zengcong@idsse.ac.cn (C.Z.); wangy@idsse.ac.cn (Y.W.); 2 College of Earth Sciences, University of Chinese Academy of Sciences, Beijing 100864, China; 3 The Shenzhen Nobel Science and Technology Service Co., Ltd., Nanshan District, Shenzhen 440305, China; zhanggen1988@163.com; 4 Division of Life Sciences, The Hong Kong University of Science and Technology, Clear Water Bay, Kowloon, Hong Kong, China; ylan@connect.ust.hk (Y.L.); sunjinsd@gmail.com (J.S.); boqianpy@ust.hk (P.-Y.Q.)

**Keywords:** *Megabalanus volcano*, transcriptome, larval settlement, neuropeptide, opsin, GPCRs

## Abstract

*Megabalanus* barnacle is one of the model organisms for marine biofouling research. However, further elucidation of molecular mechanisms underlying larval settlement has been hindered due to the lack of genomic information thus far. In the present study, cDNA libraries were constructed for cyprids, the key stage for larval settlement, and adults of *Megabalanus volcano*. After high-throughput sequencing and de novo assembly, 42,620 unigenes were obtained with a N50 value of 1532 bp. These unigenes were annotated by blasting against the NCBI non-redundant (nr), Swiss-Prot, Cluster of Orthologous Groups (COG), and Kyoto Encyclopedia of Genes and Genomes (KEGG) databases. Finally, 19,522, 15,691, 14,459, and 10,914 unigenes were identified correspondingly. There were 22,158 differentially expressed genes (DEGs) identified between two stages. Compared with the cyprid stage, 8241 unigenes were down-regulated and 13,917 unigenes were up-regulated at the adult stage. The neuroactive ligand-receptor interaction pathway (ko04080) was significantly enriched by KEGG enrichment analysis of the DEGs, suggesting that it possibly involved in larval settlement. Potential functions of three conserved allatostatin neuropeptide-receptor pairs and two light-sensitive opsin proteins were further characterized, indicating that they might regulate attachment and metamorphosis at cyprid stage. These results provided a deeper insight into the molecular mechanisms underlying larval settlement of barnacles.

## 1. Introduction

Barnacles are the only sessile marine crustacean playing important roles in costal ecosystems which mainly inhabit in intertidal to subtidal zones and are distributed all over the world [1]. As one of the major marine fouling organisms, the existence of barnacles leads to great economic losses for removing them in marine industries and navigation [2,3]. Therefore, there is significantly increasing interest in finding an efficient way to deal with biofouling by marine biologists as well as chemists.

The life cycle of the barnacle consists of two phases, free swimming planktonic phase and permanent sessile phase. Newly hatched larvae (Nauplius I) molt six times to reach a non-feeding cyprid stage, which is the last stage of planktonic phase and the most important stage for larval settlement as well. Larval settlement of the barnacle is a complicated process regulated by both external environmental factors and internal biological factors. Due to the lack of genome information, the progress of understanding the molecular mechanisms underlying barnacle larval settlement is relatively limited. For example, Clare et al., 1995, found that elevated level of the intracellular cyclic adenosine monophosphate (cAMP) significantly increased the settlement rate of cyprids [4]. Settlement-inducing protein complex (SIPC), a glycoprotein found from the adult extracts, was demonstrated to induce the conspecific larval settlement [5].

In the last few years, the rapid development of next generation sequence (NGS), especially RNA-seq, made it possible to get the transcriptome of a species in a less time- and cost-consuming way. For barnacles, Chen et al., 2011, obtained the transcriptome of *Amphibalanus amphitrite* by 454 pyrosequencing, and 23,451 contiguous sequences were identified. Among them, 7954 unigenes were differentially expressed between larval and adult stage, 743 unigenes were uniquely expressed in the larval stages, and 443 unigenes were at least 10-fold higher in larvae than adults [6]. Those specifically or highly expressed genes in larvae provided potential candidates for the mechanism studies on larval settlement. Based on the transcriptome database, the neuropeptidome of *A. amphitrite* was characterized, revealing 14 neuropeptides and peptide hormones through in silico transcriptome mining. Further real-time PCR and bioassay results showed that these neuropeptides were involved in larval settlement [7]. Moreover, He et al., 2012 and Zhang et al., 2013 discovered that the activation of the MKK3-p38 MAPK pathway might involve settlement [8,9]. These progresses demonstrated that transcriptome database could greatly facilitate the researches on the molecular mechanisms underlying barnacle larval settlement.

**Figure 1:**
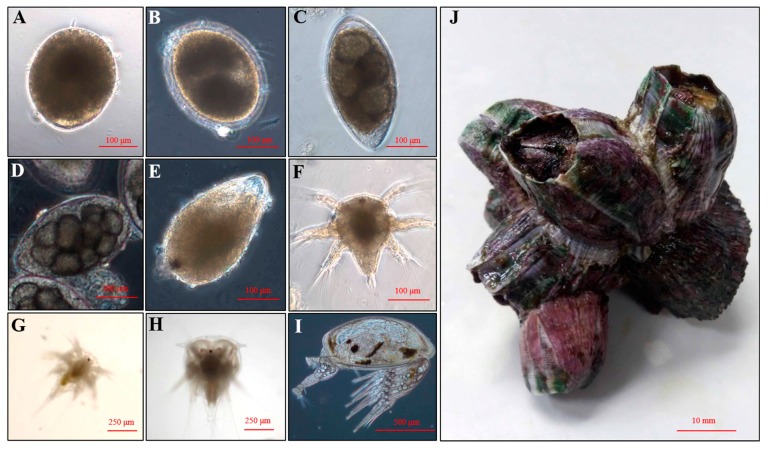
Embryos, larvae and adults of barnacle *Megabalanus volcano*. (**A**) Zygote; (**B**) 2-cells; (**C**) 4-cells; (**D**) Multi-cells; (**E**) Later developed embryo; (**F**) Fully developed embryo; (**G**) Nauplius II; (**H**) Nauplius VII; (**I**) Cyprid; and (**J**) Adult.

The cyprid of barnacle *Megabalanus volcano* (Pilsbry, 1916) is quite bigger in size than that of *A. amphitrite*, so sometimes it is a better model organism, when researchers need to observe the cyprid behavior or dissect. Yet, the genetic resources of *Megabalanus* barnacle are still lacking, which might hinder studies on molecular mechanism underlying *Megabalanus* barnacle larval settlement. In the present study, both cyprids and adults of the barnacle *M. volcano* (Figure 1I,J) were sampled for RNA-seq. The differentially expressed genes (DEGs) or pathways between cyprids and adults were identified. Neuropeptides and cognate G Protein-coupled receptors (GPCRs), and light-sensitive proteins were screened and characterized. These results provide good genomic resources of the barnacle *M. volcano* for further investigations.

## 2. Results

### 2.1. Transcriptome Sequencing, De Novo Assembly, and Unigene Annotation

Totally, 38,280,850 and 52,393,324 raw reads were generated from Illumina sequencing for cyprids and adults of *M. volcano*, respectively. Raw reads data have been submitted to the Short Read Archive (SRA) of NCBI under accession numbers of SRR5091879-50918782. After filtering the poor-quality reads, 36,661,850 (95.77%) and 50,755,042 (96.87%) clean reads were retained, respectively. All clean reads were assembled, and 55,665 transcripts were obtained. The longest one of redundant transcripts was defined as a unigene in order to reduce redundancy [10]. Finally, 42,620 unigenes were achieved, with the mean length of 865 bp and the N50 value of 1532 bp (Table 1), and the size distribution of the unigenes were shown in Figure S1. BUSCO was used to assess the completeness of the transcriptome assembly, and the results showed that 68.41% (1830 out of 2675) of the BUSCOs were complete and 10.95% (293 out of 2675) were fragmented (Table S1), indicating the good assembly quality.

Totally four public protein databases were used for blasting, and 19,666 unigenes (46.14%) had at least one hit. In details, 19,522 (45.80%), 15,691 (36.82%), 14,459 (33.93%), and 10,914 (25.61%) unigenes were annotated in the NCBI non-redundant (nr), Swiss-Prot database, Cluster of Orthologous Groups (COG) database, and Kyoto Encyclopedia of Genes and Genomes (KEGG) database. Prediction of coding regions using ESTscan showed that 7864 unannotated unigenes might have coding regions. In total, 27,530 protein coding unigenes were defined. The statistics of the Illumina sequencing data output, de novo assembly and functional annotation were summarized in Table 1.

### 2.2. Classification of Unigenes

There were 8745 out of 19,522 nr-annotated unigenes assigned to three major Gene Ontology functional categories: “Biological Process (GOBP)”, “Cellular Component (GOCC)”, and “Molecular Function (GOMF)”. In detail, 19, 18, and 13 sub-categories were in GOBP, GOCC, and GOMF, respectively (Figure S2A). In order to classify orthologous proteins, COG database was used to annotate the *M. volcano* unigenes. 14,459 unigenes were annotated and classified into 25 categories (Figure S2B). KEGG database annotation was applied to further predict the potential metabolic pathways involved. Totally, 10,914 unigenes were assigned to 226 pathways in the KEGG database. Among the 226 pathways, Ribosome (ko03010), protein processing in endoplasmic reticulum (ko04141), purine metabolism (ko00230), endocytosis (ko04144), phagosome (ko04145), RNA transport (ko03013), ubiquitin mediated proteolysis (ko04120), lysosome (ko04142), spliceosome (ko03040) and pyrimidine metabolism (ko00240) were the most abundant 10 pathways which accounted for 42.52% of all assigned unigenes.

### 2.3. Identification and Enrichment Analysis of Differentially Expressed Genes

**Figure 2:**
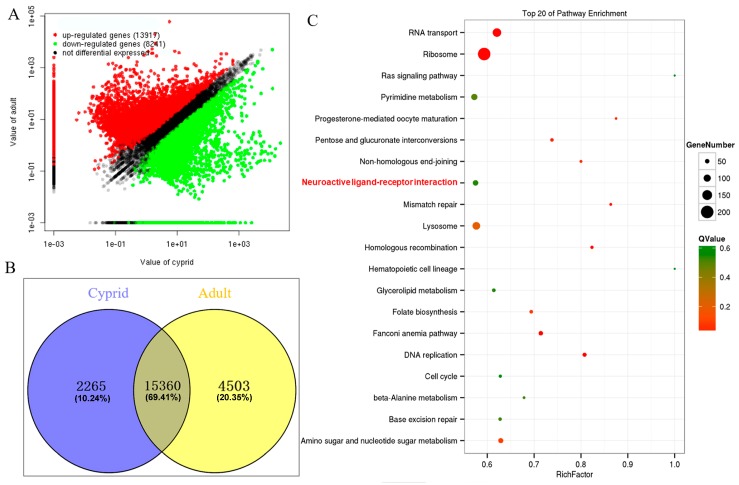
Comparison of unigene expression level between cyprid and adult stage of *M. volcano*. (**A**) Scatter diagram of unigene expression level, red and green spot indicates the differentially expressed unigenes and black indicates unigenes that were not differentially expressed between cyprid and adult stage; (**B**) Venn diagram of the differentially expressed genes (DEGs) between cyprid and adult stage; (**C**) Kyoto Encyclopedia of Genes and Genomes (KEGG) enrichment analysis of the DEGs between cyprid and adult stage.

A total of 22,158 DEGs were identified between cyprid and adult stage, with 8241 down-regulated and 13,917 up-regulated at the adult stage (Figure 2A). The reliability of the comparative transcriptomic analysis result was validated by quantitative real-time PCR analysis of eight randomly selected unigenes. It turned out that all the quantitative real-time PCR results were consistent with the result of comparative transcriptomic analysis (Figure S3). Among all the 22,158 DEGs, 2265 unigenes were exclusively expressed in cyprids and 4503 in adults (Figure 2B, Tables S2 and S3), which suggested that these two stages were quite different at the transcriptional level. Further KEGG enrichment analysis (Figure 2C) showed that the differentially expressed genes were mainly enriched in Genetic Information Processing, Metabolism, Organismal Systems and Cellular Processes classes. Notably, neuroactive ligand-receptor interaction pathway (ko04080) which belongs to Environmental Information Processing class was also enriched (*p* < 0.05). DEGs that were assigned to neuroactive ligand-receptor interaction pathway were listed in Table S4.

### 2.4. Neuropeptide Allatostatin Family and Their Putative Receptors

**Figure 3:**
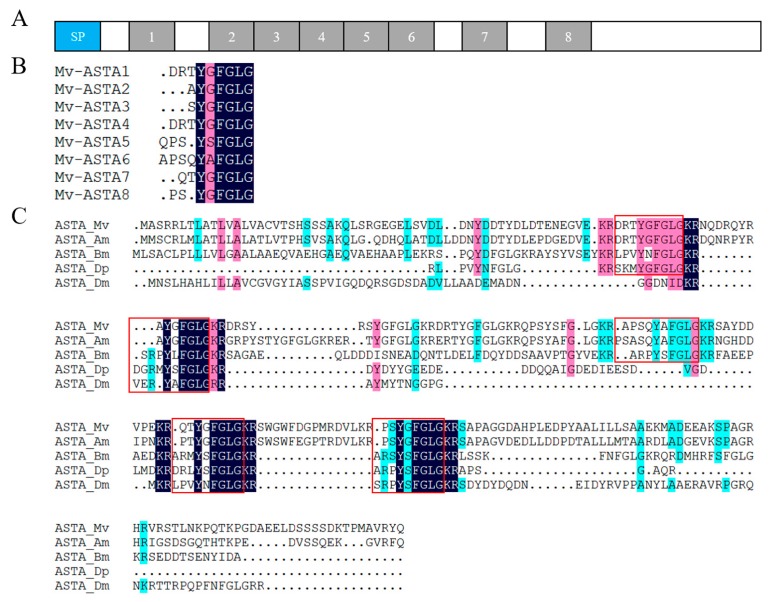
*M. volcano* A-type allatostatin (ASTA) precursor and predicted mature peptides. (**A**) Schematics of ASTA (MF579245) precursor protein, SP refers to predicted Signal peptide, 1–8 refers to predicted mature ASTA peptides; (**B**) Multiple alignment of predicted mature *M. volcano* ASTA peptides (The homology level of the sequences = 100% and ≥75% were shaded in black and pink.); (**C**) Multiple alignment of ASTA precursors from other arthropods (The homology level of the sequences =100%, ≥75% and ≥50% were shaded in black, pink and blue.), sequences in the red square are predicted mature ASTA peptides that conserved in different species (Mv: *Megabalanus volcano*, Am: *Amphibalanus amphitrite*, Bm: *Bombyx mori*, Dd: *Diploptera punctata*, Dm: *Drosophila melanogaster*).

**Figure 4:**
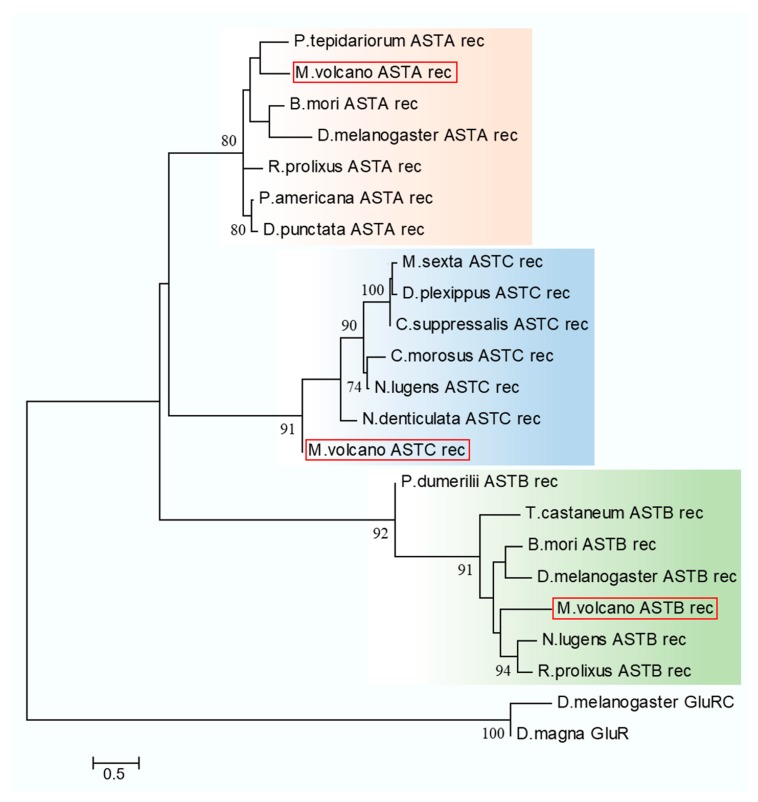
Phylogenetic analysis of allatostatin receptors. The ASTA receptors of *Megabalanus volcano* (MF579248), *Periplaneta americana* (AAK52473.1), *Diploptera punctate* (AIS40016.1), *Rhodnius prolixus* (AJA32745.1), *Parasteatoda tepidariorum* (XP_015916626.1), *Bombyx mori* (NP_001037035.1), *Drosophila melanogaster* (NP_524700.1), the ASTB receptors of *Megabalanus volcano* (MF579249), *Nilaparvata lugens* (BAO01060.1), *Bombyx mori* (NP_001108346.1), *Tribolium castaneum* (NP_001106940.1), *Rhodnius prolixus* ( AIQ81191.1), *Drosophila melanogaster* (NP_572225.1), *Platynereis dumerilii* (AFV92892.1), the ASTC receptors of *Megabalanus volcano* (partial) (MF579250), *Neocaridina denticulate* (AIY69138.1), *Nilaparvata lugens* (BAO01050.1), *Carausius morosus* (AOV81581.1), *Danaus plexippus* (EHJ63490.1), *Chilo suppressalis* (ALM88296.1), *Manduca sexta* (ADX66345.1) were used to construct the tree with Maximum Likelihood method based on LG + G model and with 100 bootstrap replications. Metabotropic glutamate receptor of *Drosophila melanogaster* (NP_001259077.1) and Glutamate receptor of *Daphnia magna* (KZS11400.1) were used to root the tree. ASTA receptors group is shaded in orange, ASTB receptors group is shaded in green, and ASTC receptors group is shaded in blue. AST receptors of barnacle *M. volcano* are labeled in the red square. Bootstrap values above 70% are indicated.

**Figure 5:**
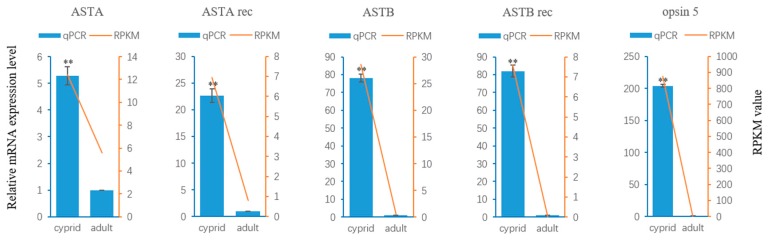
Quantitative real-time PCR analysis result of five genes. The histograms in blue show the expression level (mean ± SD) of three biological replicates, asterisks indicate significant difference detected by Student’s *t*-test (** *p* < 0.01). The line charts in orange show the reads per kilobase of unigene per million mapped reads (RPKM) values of 5 unigenes at cyprid and adult stage. ASTA, A-type allatostatin; ASTB, B-type allatostatin; ASTA rec, A-type allatostatin receptor; ASTB rec, B-type allatostatin receptor; opsin5, opsin5 gene in *M. volcano*.

**Figure 6:**
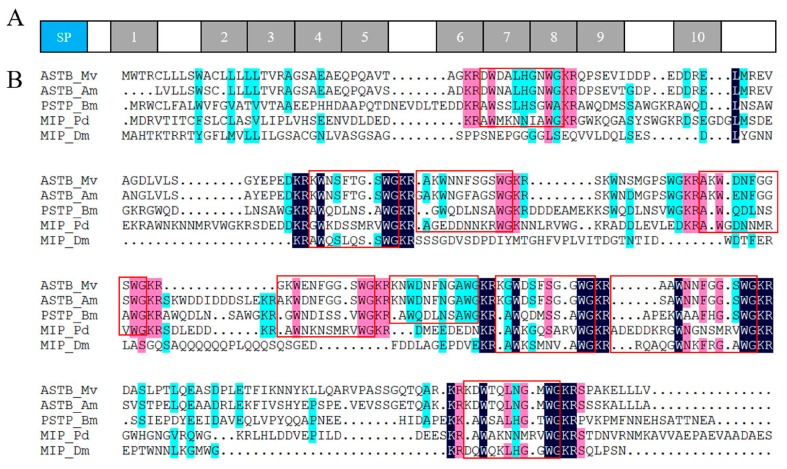

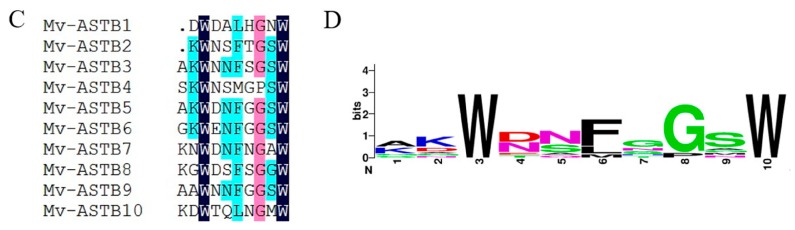
*M. volcano* ASTB precursor and predicted mature peptides. (**A**) Schematics of ASTB (MF579246) precursor protein, SP refers to predicted Signal peptide, 1-10 refers to predicted mature ASTA peptides; (**B**) Multiple alignment of ASTB precursors from arthropod and annelid (The homology level of the sequences =100%, ≥75% and ≥50% were shaded in black, pink and blue.), sequences in the red square are predicted mature ASTB peptides that conserved in different species (Mv: *Megabalanus volcano*, Am: *Amphibalanus amphitrite*, Bm: *Bombyx mori*, Pd: *Platynereis dumerilii*, Dm: *Drosophila melanogaster*); (**C**) Multiple alignment of predicted mature *M. volcano* ASTB peptides (The homology level of the sequences =100%, ≥75% and ≥50% were shaded in black, pink and blue.); (**D**) ASTB sequence logo created from an alignment of all ASTB mature peptides, ASTB5 is the peptide with the highest sequence similarity to the consensus.

Neuropeptides and cognate receptors are critical components of neuroactive ligand-receptor interaction pathway (ko04080). Here, all the three neuropeptide allatostatin (A-type allatostatin (ASTA), B-type allatostatin (ASTB), and C-type allatostatin (ASTC)) and their putative receptors were detected in the transcriptome of barnacle *M. volcano*. The precursor of *M. volcano* ASTA encoded eight mature peptides (Figure 3A), and they consisted of 6–9 amino acid (aa) residues with a common C-terminal motif YXFGLamide (Figure 3B), which is the functional characteristic of ASTA neuropeptides. ASTA precursor protein was aligned with its homologies in barnacle *A. amphitrite* and other arthropods showing that mature peptides of ASTA were conserved in different species (Figure 3C). The putative ASTA receptor was found in the barnacle *M. volcano*, which was a G protein-coupled receptor consisting of 357 aa residues. Phylogenetic analysis showed that it was closely related to those ASTA receptors that have been confirmed by experimental evidences in insects [11,12,13] (Figure 4). Both ASTA and its putative receptor (ASTA rec) had higher expression levels in cyprids than adults (Figure 5), suggesting that ASTA receptor-ligand pair might be involved in the regulation of life cycle transition and larval attachment. The precursor of *M. volcano* ASTB encoded 10 mature peptides (Figure 6A). They consisted of 9 or 10 aa residues which shared a conserved W(X_6_)W motif and were amidated (Figure 6C). ASTB5 was the most similar one to the consensus (Figure 6D). Sequence alignment of the ASTB precursor protein with its homologies from other arthropods and annelid species showed that they were highly similar (Figure 6B). One orthologue of sex peptide receptor (SPR) was identified in the transcriptome as the putative receptor of ASTB (ASTB rec). It was also a G protein-coupled receptor, and consisted of 412 aa residues. Phylogenetic analysis showed that it was well clustered together with those SPRs that have been confirmed to be the receptors of corresponding ASTB [14,15,16,17] (Figure 4). Both ASTB and its putative receptor (ASTB rec) were down-regulated at adult stage (Figure 5), suggesting that ASTB peptides might regulate larval attachment and metamorphosis at cyprid stage. ASTC and its putative receptor ASTC rec were also identified in the transcriptome of barnacle *M. volcano* (Figure S4 and Figure 4), but both of them had a very low expression level and had no significant difference between cyprids and adults.

### 2.5. Light-Sensitive Proteins

**Figure 7:**
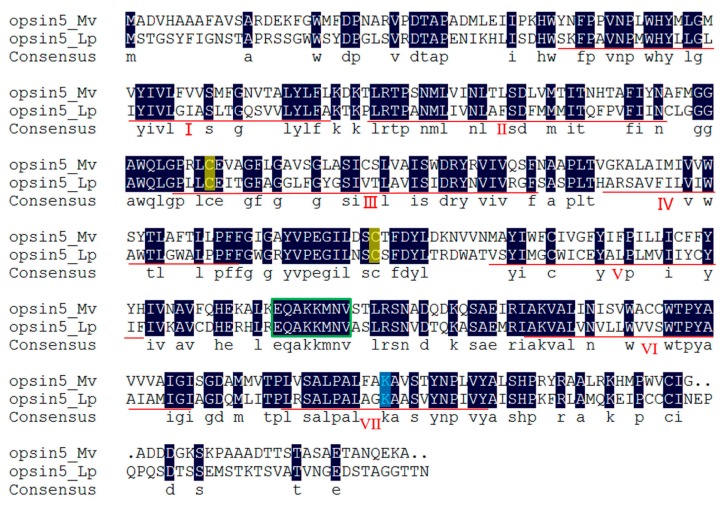
Sequence alignment of *M. volcano* opsin5 (MF579251) (opsin5_Mv) with that of *Limulus polyphemus* (ACO05013.1) (opsin5_Lp). The homology level of the sequences = 100% were shaded in black. Seven transmembrane domains were underlined and labeled from I to VII; two Cys (C) residues conserved in opsins were highlighted in yellow; the conserved Lys (K) residue in red is critical for schiff base formation with the chromophore; sequences in green square were highly conserved in arthropod opsins.

**Figure 8:**
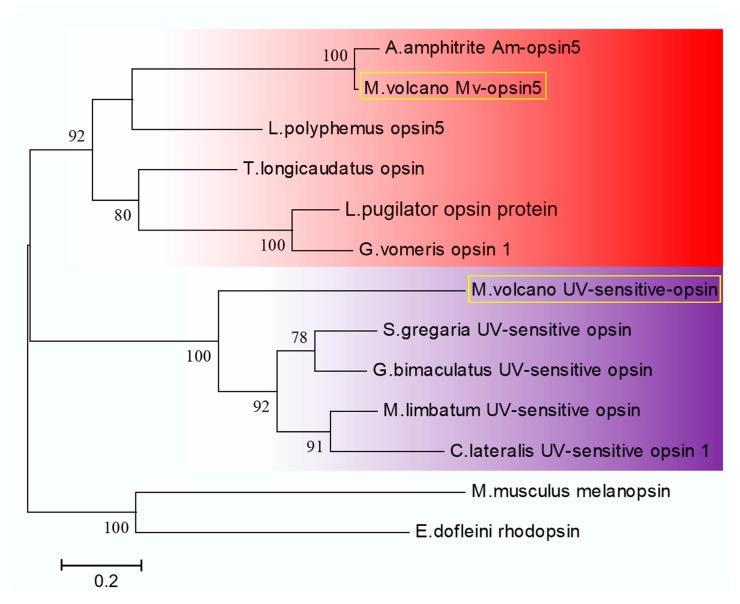
Phylogenetic analysis of light-sensitive opsin proteins. The opsin5 of *Megabalanus volcano* (MF579251), opsin5 of *Amphibalanus amphitrite*, opsin protein of *Leptuca pugilator* (ADQ01810.1), opsin1 of *Gelasimus vomeris* (ACT31580.1), opsin of *Triops longicaudatus* (BAG80981.1), opsin5 of *Limulus polyphemus* (ACO05013.1), the UV-sensitive opsin of *Megabalanus volcano* (MF579252), *Schistocerca gregaria* (BAP16681.1), *Gryllus bimaculatus* (AEG78686.1), *Macrosiagon limbatum* (APY20510.1) and *Chrysobothris lateralis* (ANN11830.1) were used to construct the tree with Maximum Likelihood method based on LG+G model and with 100 bootstrap replications. Melanopsin of *Mus musculus* (NP_038915.1) and rhodopsin of *Enteroctopus dofleini* (X07797.1) were used to root the tree. Long wavelength-sensitive opsin group is shaded in red, UV-sensitive opsin group is shaded in purple. Light-sensitive opsin proteins of barnacle *M*. *volcano* are labeled in the yellow square. Bootstrap values above 70% are indicated.

Two light-sensitive proteins that were also G protein-coupled receptors were identified in the transcriptome of *M. volcano*. One had the highest similarity to *Limulus polyphemus* opsin5 which is sensitive to visible light (400–700 nm), and thus was named Mv-opsin5. Both comparative transcriptomic analysis and quantitative real-time PCR results showed higher expression level of Mv-opsin5 gene in cyprids, in comparison to adults (Figure 5). The putative amino acid sequence of Mv-opsin5 in *M. volcano* was aligned with the coding sequence of *L. polyphemus* opsin5, and the results showed that opsin5 in *M. volcano* had the typical features of rhabdomeral opsins [18], including seven predicted transmembrane domains, two conserved cysteine residues at C^129^ and C^206^, a conserved lysine (K^327^) in transmembrane helix VII which is essential for the schiff base binding of the chromophore and a string of eight aa (E^256^ to V^263^) in cytoplasmic loop 3 (Figure 7). Furthermore, Mv-opsin5 was clustered together with long wavelength-sensitive opsins of other arthropods in phylogenetic analysis, indicating that Mv-opsin5 might be sensitive to long-wavelength light (Figure 8). The other light-sensitive protein had the highest similarity to *Schistocerca gregaria* UV-sensitive opsin, and was named Mv-UVS-opsin. Notably, it was exclusively expressed in cyprids and has almost no expression in adult according to the RPKM value (Table S2). Phylogenetic analysis showed that it fell in the same group with other arthropod UV-sensitive opsins [19,20,21] (Figure 8), suggesting that it might be sensitive to ultraviolet.

## 3. Discussion

The barnacle *M. volcano* is one of the major fouling species in the South China Sea [22]. Here, we presented the transcriptome of *M. volcano* using RNA-seq and de novo assembly. Up to date, the present study is the only transcriptome information of *Megabalanus* barnacle, which might support the biofouling researches on this genus in the future.

In this transcriptome, a total of 22,158 (51.99%) DEGs were identified through comparative transcriptomic analysis, indicating that cyprids and adults are quite different at the transcriptional level. It was in accordance with the fact that they are morphologically distinct because of metamorphosis and occupying different ecological niche [6]. 8241 unigenes that had higher expression level in cyprids than adults suggested that they may play more important roles at cyprid stage, the key stage preparing for attachment and metamorphosis. KEGG enrichment analysis of the DEGs identified 17 significant enriched pathways, undoubtedly, they primarily participate in DNA replication and repair, translation, substantial and energy metabolism to sustain the fundamental requirement for the complicated life cycle transition. In addition, neuroactive ligand-receptor interaction pathway (ko04080) belonging to Environmental Information Processing class was also enriched. It is related to the fact that barnacles at cyprid and adult stage live in different environmental conditions. Since environmental cues are indispensable instructors during life cycle transitions and life behaviors in many marine invertebrates [16,23,24], and certain neuroactive substances have been proven to influence barnacle cyprid settlement behavior [25], so that it was reasonable that neuroactive ligand-receptor interaction pathway might participate in barnacle larval settlement regulation.

Neuropeptides are crucial neuroactive ligands that belong to the neuroactive ligand-receptor interaction pathway (ko04080). They regulate numerous physiological processes like development, reproduction, courtship, feeding, and metabolism in insects by binding to their corresponding receptors [26]. As neuropeptide ASTA, ASTB, and their putative receptors were significantly down-regulated in adults compared with the cyprid stage, these neuropeptides might play important roles at the cyprid stage. One of the major functions of ASTs in insects is to inhibit juvenile hormone (JH) synthesis [27,28]. In barnacles, physiologically-relevant concentration of methyl farnesoate which served as a JH restrained larval settlement although exogenous methyl farnesoate in high concentration inspired precocious metamorphosis [29,30]. Previous research has deduced that AST peptides were likely to have the ability to suppress JH synthesis in the barnacle *A. Amphitrite* [7], which might be applied in *M. volcano* as well. Cyprid is a non-feeding stage, and during this stage, energy mainly comes from the lipids in oil cells [31,32,33]. In *Drosophila,* ASTA can accelerate the usage of lipids and regulate metabolism and feeding decision [34]. In the present study, we speculated that a high expression level of ASTA at the cyprid stage might also be necessary for the regulation of the usage of lipids and inhibition of feeding decision. ASTC and its putative receptor were identified in the transcriptome of *M. volcano*, but they had a very low expression level and had no significant difference between cyprid and adult stages. In addition, ASTC had the highest expression level at nauplius VI stage of *A. Amphitrite* [7], suggesting that they probably functioned at nauplius stages rather than at the cyprid or adult stage.

Barnacles at the cyprid stage have well evolved nervous system to detect specific environmental cues that mediating settlement [25], of which, a median eye and two compound eyes are served as photoreceptors, but their roles are not well elucidated yet [35]. Opsins are a group of light-sensitive proteins mediating the transition of photons of light to electrochemical signal which is the first step of visual transduction cascade. The current evidence in *M. volcano* indicated that Mv-opsin5 was thought to be sensitive to long-wave length light, moreover, it clustered well with Am-opsin5 (Figure 8) and was highly expressed at the cyprid stage (Figure 5), suggesting that cyprids of *M. volcano* might depend on Mv-opsin5 to discriminate color and prefer to settle on red surfaces as *A. Amphitrite* [18,36]. Many marine zooplankton species show synchronized circadian movement defined as diel vertical migration (DVM) [37,38,39], so do barnacle larvae, although the patterns might differ among different stages and species [40,41]. One of the roles of DVM is to keep the zooplankton away from UV damage and predation [39,42]. Therefore, we speculated that the planktonic larvae of barnacles probably utilize the UV-sensitive opsin to detect ambient UV signals from sunlight, and further adjust their DVM to avoid UV damage and predation.

G protein-coupled receptors (GPCRs) are a large family of seven-transmembrane integral membrane proteins that mediate the signal transduction from the outside to the inside of cells. In humans, GPCRs are involved in a lot of diseases and they are often the targets of many pharmaceutical drugs [43], and approximately 40% of all the modern medicinal drugs target GPCRs [44]. In insects, GPCRs play important roles in modulating a great many physiological activities and behaviors, including reproduction, growth, development, etc. Modifying the normal function of given GPCRs by disturbing their actions would cause unfitness or even death to a pest, implying that GPCRs can offer potential targets for the development of next generation pesticides [45]. As the major marine biofouling organism, barnacles have drawn lots of attention from pharmacologists, and several compounds have been found to inhibit the settlement of *Balanus improvises* by interacting with α-adrenoceptor [46,47]. From this point, GPCRs might also be potential targets for the development of antifouling compounds. Agonist or antagonist against GPCRs that play important roles in life stage transformation and larval attachment (such as ASTA receptor, ASTB receptor, Mv-opsin5, and Mv-UVS-opsin) might be designed to obstruct their normal functions. Therefore, further investigations are necessary to better understand the characteristic and functions of GPCRs in barnacles.

## 4. Materials and Methods

### 4.1. Ethics Statement

The barnacle *M. volcano* is a common biofouling species which is not endangered or protected. Adult barnacles of *M. volcano* used in this research were collected from populations growing on the rocky shore at Shek O Country Park in Hong Kong (22°13′41″ N, 114°15′22″ E) which does not belong to any national parks, protected areas, or privately owned places, and needs no specific permits for adult barnacle collecting. The field studies did not involve any endangered or protected species.

### 4.2. Larval Culture

Adult barnacles of *M. volcano* were taken down from the rocky shore without hurting the base plates. They were cleaned thoroughly with a brush and washed with 0.22 μm-filtered sea water (FSW). The adult barnacles were dried in the air for 24 h at 25 °C, after then they were transferred into FSW. Embryos at different developmental stage (Figure 1A–F) were released instead of swimming larvae. The embryos were collected and hatched in FSW at 28 °C for 1–2 h until later developed and fully developed embryos developed into nauplius stage (Figure 1G). After that, all the nauplii were transferred into another tank and cultured according to the procedure of a common procedure [48]. Briefly, larvae were maintained at a density of 1 larva mL^−1^ in autoclaved FSW at 25 °C with the light:dark cycle of 12 h:12 h. The larvae were fed with *Chaetoceros gracilis* at about 1 × 10^6^ cells/mL every day until they transformed to cyprid stage. They took 8–10 days to transform from nauplius to cyprid stage.

### 4.3. RNA Extraction, RNA-Seq Library Construction and Sequencing

Total RNA of three pools of cyprids (15 cyprids for each pool) and three adult barnacle *M. volcano* were extracted with Trizol Reagent (Invitrogen, Waltham, MA, USA) following the manufacturer’s instructions. The quality and quantity of RNA were verified by RNAase-free agarose gel and Agilent Bioanalyzer 2100 system (Agilent Technologies, Santa Clara, CA, USA). The three RNA samples for cyprid and three RNA samples for adult barnacle were pooled respectively for library constructing. The mRNA was isolated with oligo-dT beads (Qiagen, Hilden, Germany), and then sequencing libraries were constructed using NEBNext^®^ UltraTM RNA Library Prep Kit for Illumina^®^ (NEB, Ipswich, MA, USA) according to manufacturer’s instructions. High-throughput sequencing was performed using Illumina HiSeq™ 4000 platform (Illumina Inc., San Diego, CA, USA) in paired-end mode with the read length of 125 bp after the library quality was assessed by Agilent Bioanalyzer 2100 system.

### 4.4. De Novo Assembly and Functional Annotation

Raw reads generated were filtered by removing reads with adaptor sequences, or with more than 10% unknown nucleotides, or with more than 40% low quality bases (base quality ≤ 20) to obtain high-quality clean reads. All the following analyses were based on clean reads. Clean reads of cyprids and adults were pooled and used for de novo assembly by Trinity algorithm^2.0.6^ [49]. BUSCO^v2^ was used to assess the transcriptome assembly completeness [50]. Unigenes obtained from the output of Trinity were searched against public protein databases including NCBI non-redundant (nr) database, Swiss-Prot, COG and Kyoto Encyclopedia of Genes and Genomes (KEGG) (e-value < 0.00001) using BLASTx^2.2.29+^ [51]. Unigenes that had no hits to any databases were used to predict coding regions and figure out the sequence direction by ESTScan^3.0.2^ [52]. Gene Ontology annotation was analyzed by Blast2go [53], following functional classification by WEGO [54].

### 4.5. Differential Gene Expression Analysis

Clean reads from cyprids and adults were mapped to the assembled whole transcriptome with software bowtie2. The number of mapped reads to every unigene was counted using SAMtools. Unigene expression level was quantified with RPKM (Reads Per Kilobase of unigene per Million mapped reads) method [55], and unigenes that RPKM value ≥0.3 was defined as expressed [56]. Differentially expressed genes were analyzed by edgeR with the threshold FDR ≤ 0.05 (False Discovery Rate, is a statistical method used in multiple hypothesis testing to correct for *p*-value [57]) and |log_2_Fold Change| ≥ 1. KEGG enrichment analysis was performed using cumulative hypergeometric distribution as the method used for *Bathymodiolus platifrons* [58]. Enrichment for a KEGG pathway was determined based on the *p* value ≤ 0.05.

### 4.6. Validation of RNA-Seq Results by Quantitative Real-Time PCR

Quantitative Real-time PCR was performed with KAPA SYBR^®^ FAST qPCR Kit (Kapa Biosystems, Wilmington, MA, USA) using the StepOnePlus^TM^ Real-time PCR system (Applied Biosystems) according to the standard protocol. Total RNA was extracted with Trizol Reagent (Invitrogen, Waltham, MA, USA) as indicated above, and High Capacity cDNA Reverse Transcription Kits (Applied Biosystems, Waltham, MA, USA) was used for 1st strand cDNA synthesis. Gene specific primes (Table S5) were designed with Primer-Blast (https://www.ncbi.nlm.nih.gov/tools/primer-blast/). The qPCR reaction volume was 20 μL containing 7.8 μL PCR-grade water, 10 μL Mix, 0.4 μL ROX and 0.4 μL Forward Primer, 0.4 μL Reverse Primer, 1 μL template cDNA in the final concentrations of 0.2 μM, 0.2 μM and 0.5 ng/μL respectively. PCR was performed with a program of 95 °C for 3 min, followed by 40 cycles of 95 °C for 2 s and 60 °C for 20 s. Three biological replicates and three technical replicates were performed for each gene, 2^−ΔΔ*C*t^ method [59] was utilized to evaluate the relative expression level of the genes with *actin* as the internal control.

### 4.7. Sequence Alignment and Phylogenetic Analysis

Sequence alignment was performed with Clustal X 2.1 (EMBL, Heidelberg, Germany) and shaded with DNAMAN 8.0 (Lynnon Biosoft, San Ramon, CA, USA). Phylogenetic tree was constructed with MEGA 7.0 in a Maximum Likelihood method with the amino acid sequence of the proteins, branch support values were estimated from 100 bootstrap replicates with default settings [60].

## Figures and Tables

**Table 1:** Summary of assembly and annotation of *Megabalanus volcano* transcriptome.

Results	*Megabalanus volcano*
**Output result**	
Raw reads	90,674,174
Clean reads	87,416,892 (96.41%)
**Assembly result**	
Unigene number	42,620
Unigene mean length (nt)	865
Unigene N50 (nt)	1532
**Annotation result**	
Nr	19,522
SwissProt	15,691
COG	14,459
KEGG	10,914
Gene Ontology	8745
**Coding sequence prediction**	
CDS predicted from BLAST result	19,666
CDS predicted by ESTscan	7864
Total CDS predicted	27,530

## Supplementary Materials

Supplementary materials can be found at www.mdpi.com/1422-0067/18/11/2253/s1.
