# Supplementary material for: Comparative Transcriptomic Analysis Reveals Candidate Genes and Pathways Involved in Larval Settlement of the Barnacle Megabalanus volcano
Source: Int J Mol Sci. 2017 Oct 27;18(11):2253. doi: 10.3390/ijms18112253 (PMC5713223; doi:10.3390/ijms18112253)
Supplement: Supplementary file 1 [file ijms-18-02253-s001.zip › Supplementary Information--FigureS1-S4.pdf]

Supplementary Information

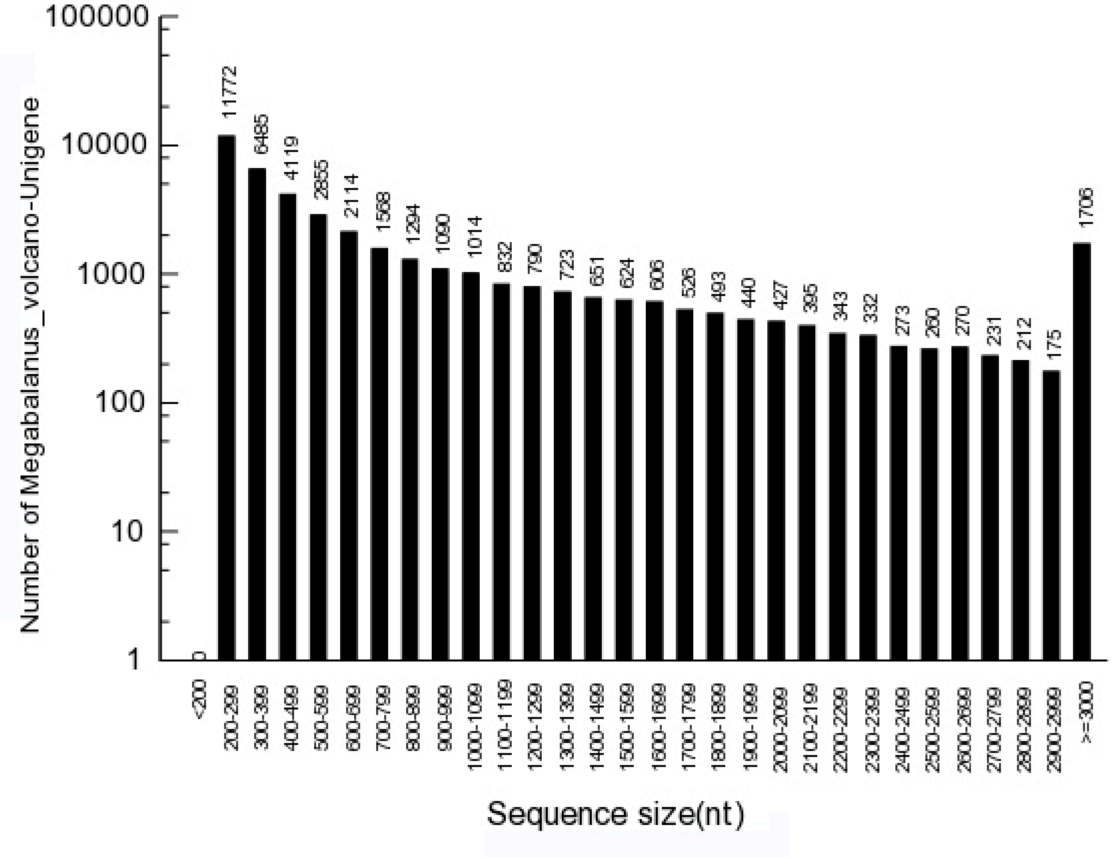

Figure S1. Size distribution of *M. volcano* unigenes.

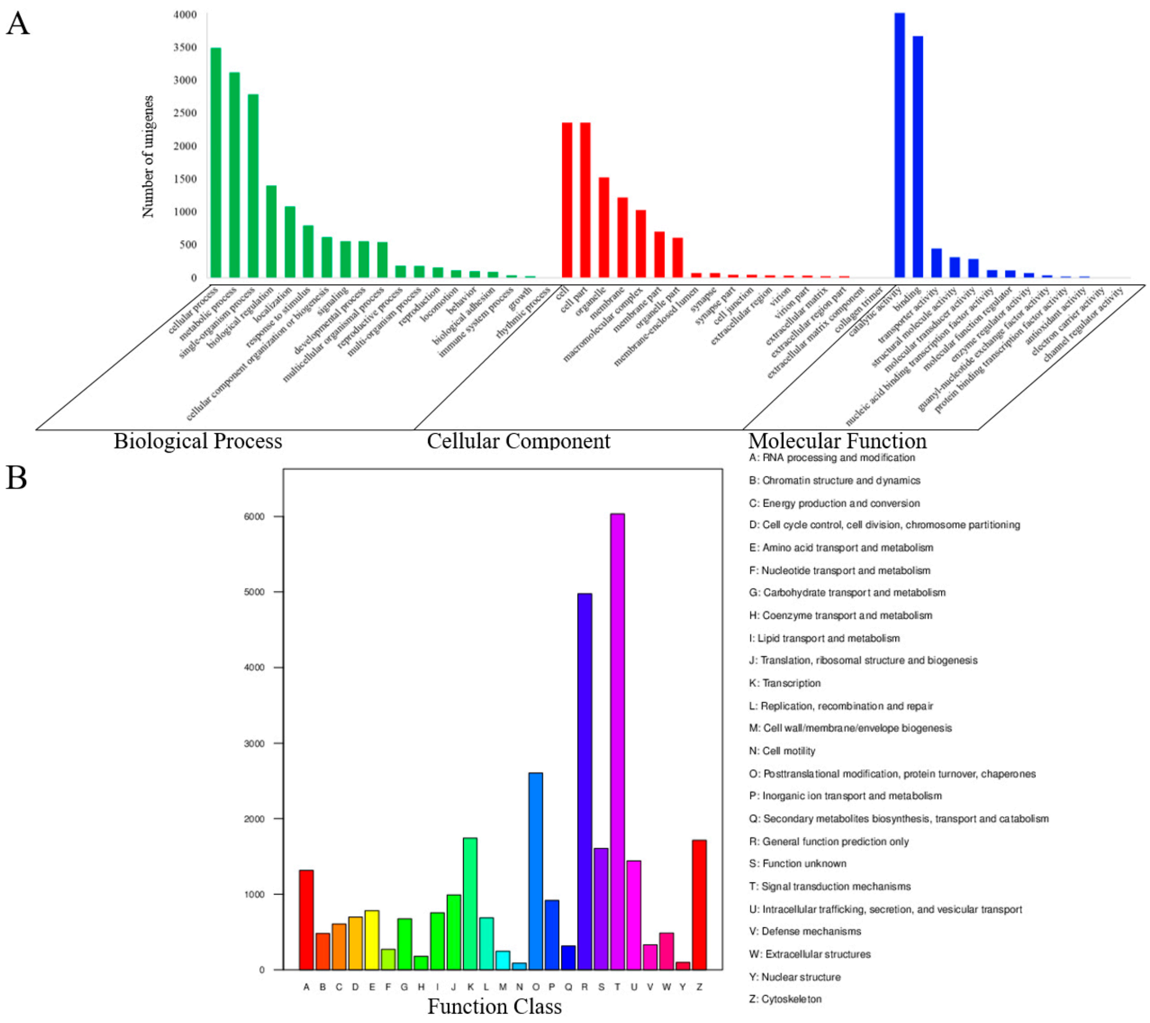

Figure S2. Classification of unigenes in *M. volcano* transcriptome. A. Functional classification of annotated unigenes by Gene Ontology, B. Functional classification of annotated unigenes by COG.

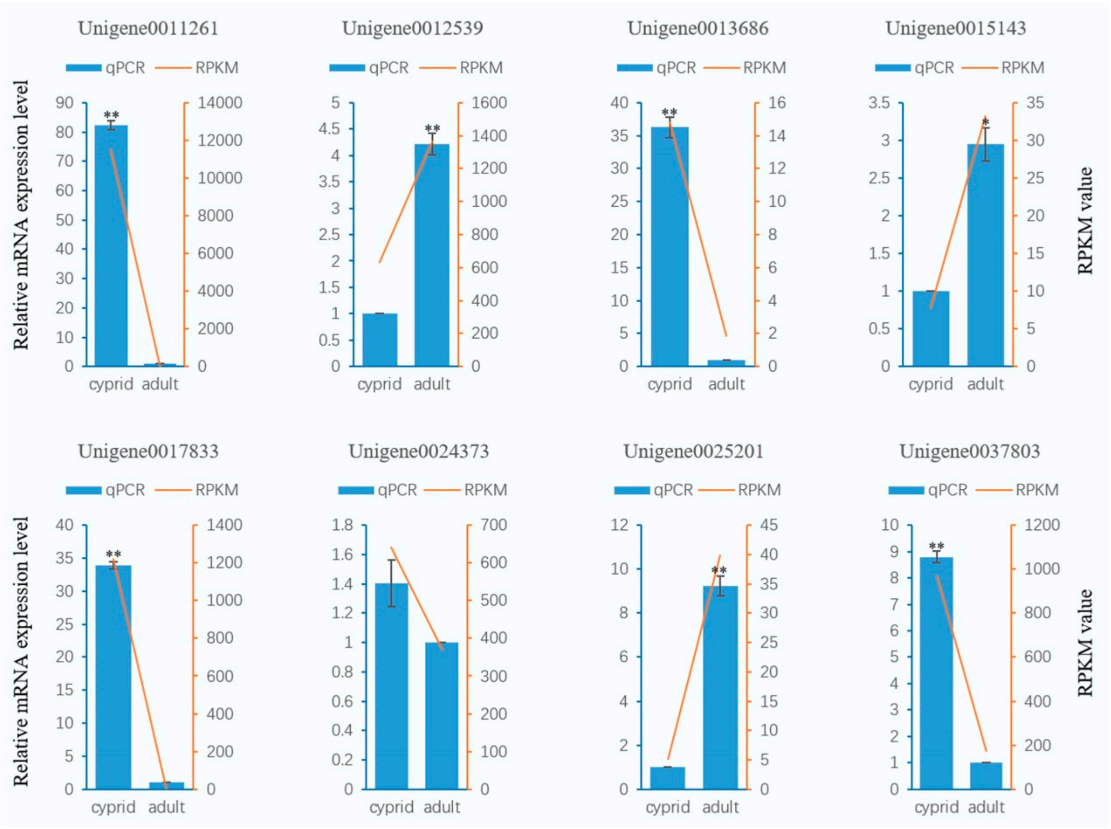

Figure S3. Quantitative Real-time PCR validation of eight randomly selected genes. The histograms in blue show the expression level (mean ±SD) of three biological replicates, Asterisks indicate significant difference detected by Student's t test (* $p < 0.05$, ** $p < 0.01$). The line charts in orange show the RPKM values of 10 unigenes at cyprid and adult stage.

1 ATGACGCTGGCCCGCCTCACACTGCTGCTGGCGCTCTGTCTGGCC
1 M T L A R L T L L L A L C L A
46 ACTCTTCTCTCTTCCGTGGCCTCCAAGAGCCTCCAGAAGGACGTC
16 T L L S S V A S K S L Q K D V
91 TTCTCGCCCCACTACGAGGGCGAGGACCACAAACTGGACGTGATG
31 F S P H Y E G E D H K L D V M
136 CCAGACGACGGGACGGCCGAGACGGCGCTGCTCAACTATTTGTAC
46 P D D G T A E T A L L N Y L Y
181 GCCCGTCAGATGTTGCGCCGTCTGAGCAACAATCTGGACGTCACC
61 A R Q M L R R L S N N L D V T
226 GAACTGCAGCGCAAGCGCTCCTACTGGAAACAGTGCTCCTTCAAC
76 E L Q R K R S Y W K Q C S F N
271 GCCGTCTCCTGCTTCGGCTAA
91 A V S C F G *

Figure S4. *M. volcano* ASTC (MF579247) precursor and predicted mature peptide. Signal peptides are marked as green letter, precursor related peptides are marked as blue letter, and mature neuropeptide is marked as red letter. Possible cleavage sites are underlined.
